# Tree Value Visions – integrating relational values and multispecies justice in urban treescape management

**DOI:** 10.1038/s42949-026-00410-4

**Published:** 2026-07-13

**Authors:** Jasper O. Kenter, Peter Wood, Alison Dyke, Angelika Zimmermann, Joanne Morris, Janice Ansine, Austin Himes, Barbara Muraca, Liz O’Brien, Holly Woo

**Affiliations:** 1 Aberystwyth Business School, Aberystwyth University, Aberystwyth, UK; 2 Ecologos Research Ltd., Aberystwyth, UK; 3 Department of Environment and Geography, University of York, York, UK; 4 School of Social Science and Global Studies, The Open University, Edinburgh, UK; 5 Stockholm Environment Institute, University of York, York, UK; 6 Loughborough Business School, Loughborough University, Loughborough, UK; 7 School of Environment, Earth and Ecosystem Sciences, The Open University, Milton Keynes, UK; 8 School of the Environment, College of Agricultural, Human and Natural Resources Sciences, Washington State University, Pullman, USA; 9 Department of Philosophy & Environmental Studies Program, University of Oregon, Eugene, USA; 10 Forest Research, Alice Holt Lodge, Farnham, UK

**Keywords:** Ecology, Ecology, Environmental social sciences, Environmental studies, Geography, Geography

## Abstract

Urban treescapes are important nature-based solutions for climate resilience, biodiversity, and human wellbeing, but their management often marginalizes relational values and multispecies justice. Tree Value Visions is a low-resource deliberative tool for inclusive, values-led urban treescape planning, grounded in the IPBES Life Framework. It facilitates community-driven visioning, linking storytelling to prioritization of outcomes and practical actions. Case studies suggest the tool can successfully foster synergies between human and ecological needs.

## Introduction

Expansion of treescapes is recognised as a key nature-based solution in international and national recommendations to reduce net carbon emissions^1^. For example, in the United Kingdom (UK), the national Climate Change Committee^2^ has established tree-planting targets of 30,000 hectares per year. Local authorities must consider how to realise these targets in their areas, and also how to meet Sustainable Development Goal 11 on making cities inclusive, safe, resilient and sustainable^3^. Treescapes in urban areas are thus likely to see substantial change. These changes must balance many, often competing, policy objectives, such as around carbon, biodiversity, flood regulation, local climate regulation, and physical and mental health, with the space and resource constraints of urban areas and local authorities. Achieving this requires evidence-led decision making on nature-based solutions by planners and developers.

Increasingly, such decisions include assessments and valuations of ecosystems. Treescapes - all the trees within a local area and the green spaces associated with them, such as street trees, parks and urban nature reserves^4^ - are known to be valuable assets. However, in practice, valuation and policy decisions have mostly focused on relatively well understood and readily quantified benefits, such as carbon storage and amenity value of trees. Moreover, management approaches are predominantly technocratic and heavily focused on practical management considerations (safety, infrastructure, capacity, etc.), with often only limited community participation^5^. A limit of such approaches is that they do not adequately consider relational values: the importance of meaningful, often reciprocal, human relationships with nature, and among people through nature^6^. One reason for this limitation is that relational values are often based on identities and emotions^7–9^, which do not sit easily within dominant technical valuation languages. Therefore, new tools and approaches that better recognise and reflect relational discourses of value are required to more fully illicit the importance of treescapes^7,10,11^.

Besides being technocratic, with the exception of some legal measures that recognise nature’s intrinsic value (e.g., the European Union’s 1992 Habitats Directive), dominant valuation and management languages in urban planning and development have also mostly been anthropocentric^12^. Justice, if considered from this perspective, typically refers to fair distribution of benefits and risks between people, equitable access to natural resources such as amenity spaces, and fair representation and process for people that are affected by decisions. In contrast, Multispecies Justice (MSJ) comprises theories of justice that recognise the interests of non-human entities such as animals, plants, and ecological systems like forests and rivers. MSJ establishes a moral obligation for societal institutions, including political and legal systems, to consider them when making decisions^13^. Institutional anthropocentrism makes it challenging to expand concepts of justice beyond humans in urban treescape planning^12^. There is thus a need for new tools that can help implement new treescape policies whilst better integrating both more diverse values, and the considerations and justice claims of local human and more-than-human communities.

Tree Value Visions^14^ is a practical tool that integrates community-based visioning of future treescapes with a process for prioritising outcomes and policy actions. In doing so, it seeks to integrate relational values and do justice to the needs of both local communities of people and the justice claims of non-humans. An important way in which we can identify the justice claims of non-humans is through recognising their intrinsic values and articulating their needs (for example, trees need space for root growth)^15,16^. By identifying what non-humans need to flourish, established theories of justice such as the capability approach or Rawlsian frameworks can be extended^17,18^. However, ethical claims can also be grounded in relational values emphasising empathy, care, kinship, reciprocity, heritage, place and harmony in relationships between people and nature. Such relational values are often grounded in lived experience and embodiment^11,19^. They can underpin rights or legal personhood of non-human entities but can also be expressed through ethics of care^20,21^.

Consequently, more explicit recognition of relational values could benefit MSJ by providing a further axiological basis for justice in addition to the intrinsic worth of non-humans. Some advocates of the salience of relational values have argued that, due to their connection to lived experience, these values may bear more direct relevance and experiential meaning to communities than more abstract ethical arguments for nature conservation centred around intrinsic values^22^. Some empirical work suggests that intrinsic and relational (and also some instrumental) values are frequently ‘layered’ in the way people articulate nature's values and are thus co-emergent in real-life advocacy for nature^6,16^.

**Fig. 1:**
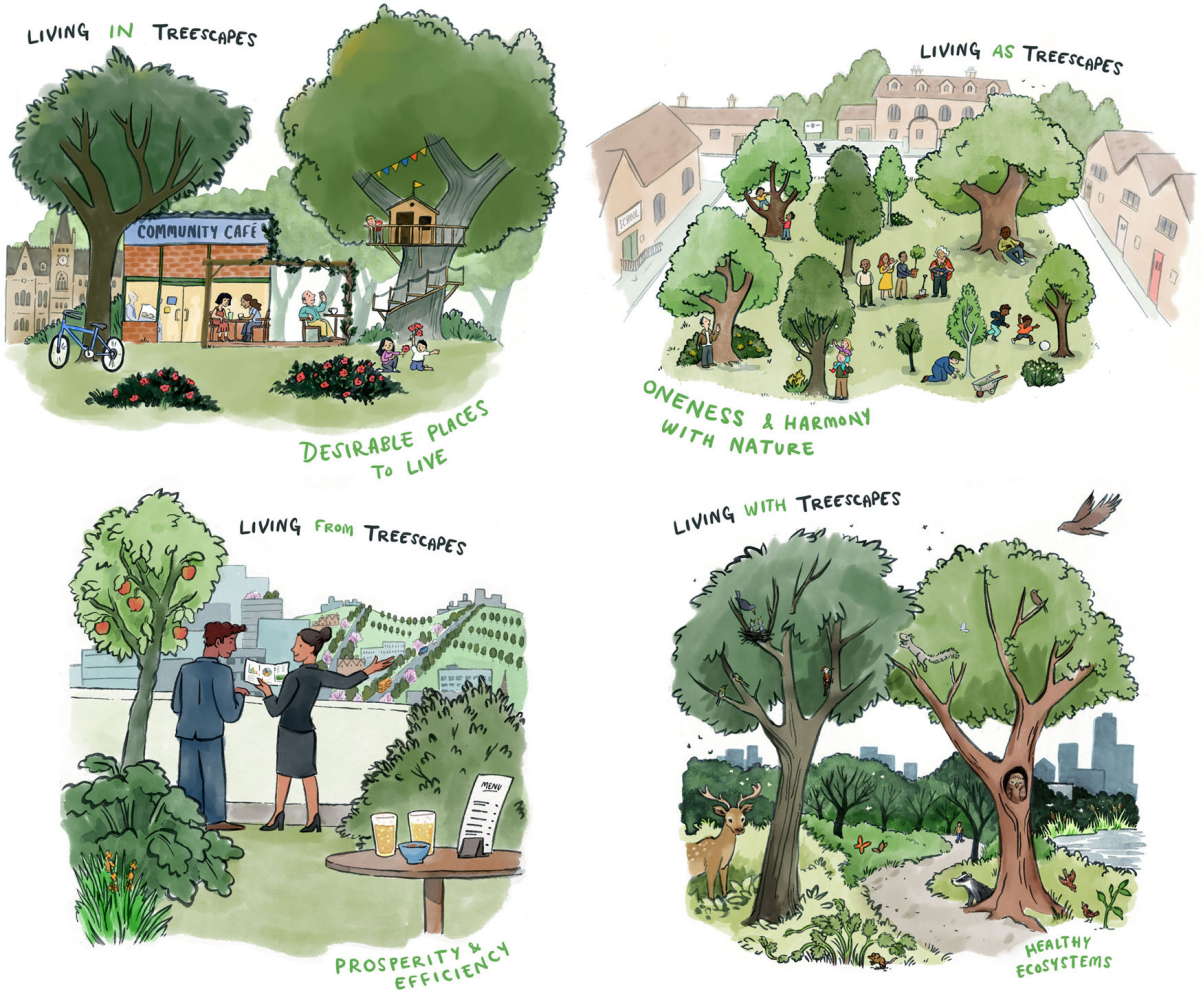
Visual representations of the four treescape visions, with their focal broad values. The supplementary material displays the images alongside the relevant vision text. Artwork by Helen Panayi.

Tree Value Visions layers pluralistic values within four value-based future visions (Fig. 1). It consists of a set of four template urban treescape visions, comprising text, audio-recording, and images, and a template low-resource participatory process. Practically, the tool seeks to support local deliberative democratic decision-making with urban residents or treescape stakeholders, within contexts of resource constrained local governance. Tree Value Visions supports identifying priorities across diverse relational, intrinsic, and instrumental treescape values, and policy and community actions for operationalizing these values and diverse modes of human-nature relating. Many of these actions reflect practical ways of operationalising MSJ, from making more space for nature in cities to new rights for non-humans, to actions that encourage oneness and harmony between people and treescapes, such as policies to engage children with nature.

The visions are grounded in the Life Framework^16,23^ adopted by the Intergovernmental science-policy Platform on Biodiversity and Ecosystem Services (IPBES) to organize its typology for nature’s values^6,10^. Four Life Frames– living *from*, living *in*, living *with*, and living *as* nature - can be seen as lenses that characterise how people, policies and institutions emphasize different sets of values associated with certain ways of being, living and relating to nature^5,24^. Tree Value Visions is one of the first practical tools to apply this framework. Training in the tool is supported by an open access, self-directed online course^14^.

We first introduce Tree Value Visions in its background, approach, and potential uses by local actors. We subsequently illustrate application of the tool through a case study of five UK cities: Cardiff, Milton Keynes, Edinburgh, York, and Camden (a borough of London), with particular attention to how Tree Value Visions integrates MSJ and relational value considerations within its process and outcomes.

### About tree Value Visions

Tree Value Visions is a group deliberative tool focused on rapidly forming and identifying value priorities with participants. Deliberation participants can be urban residents or local community stakeholders of limited technical expertise. Its primary intended ‘user-group’ is of local treescape decision-makers, such as local authority tree officers or planning departments, third sector organisations that manage or influence the development urban greenspaces (e.g., park trusts, community land trusts), or high technical-expertise community stakeholders.

Tree Value Visions includes the following main components:

1. Four archetypal narrative visions of the future centred around the four Life Frames (Fig. 1): *living in treescapes*: trees contributing to desirable places to live, work and recreate; 2) *living from treescapes*: trees as a resource, emphasising prosperity and efficiency; 3) *living as treescapes*: trees being part of the community, emphasizing oneness and harmony with trees and treescapes (Box 1); 4) *living with treescapes*: trees and treescapes as space for nature, emphasising healthy ecosystems (Box 2). The full set of visions is presented in the Supplementary Material (Text S1).
2. A set of fifteen high level potentially desirable outcomes of future treescapes, divided in outcomes relating to community, to nature and environment, and to transport, economy, and development (Table 1).

**Table 1:** Possible outcomes of future visions that participants are asked to prioritise

Outcome	Description	Most central in
**Community**		
1. Community participation and cohesion	Local communities can participate more in decisions and people within communities become more close-knit and connected.	Living in, living as
2. Equity in distribution and access to trees and greenspaces	Access to nature becomes more equal across the city and across different social groups.	Living in
3. Health and wellbeing	People are mentally and physically healthier and experience greater wellbeing.	Living in, living as
4. Trees as community members	Trees are a more integral and felt part of the community and treated more as community members with their own lives and needs that are deemed important to consider.	Living as
5. Tree heritage	The history, heritage, and cultural value associated with trees is protected and increased.	Living in
**Nature and environment**		
6. Abundance of natural life in the city	There are more plants and animals in the city.	All
7. Protection of endangered species	Species that are rare and threatened have more access to suitable habitat and food, and are better protected.	Living with
8. Resilience to climate change	People and wildlife are better protected from the effects of climate change, including extreme weather such as heatwaves, droughts, extreme rainfall and floods.	Living with
9. Clean air and water	Air and water quality is better regulated by trees, with pollution reduced.	Living with
10. Education and awareness about trees and nature	People better understand trees, nature and greenspaces, and their values.	Living as
**Transport, economy and development**		
11. Space for cars	A similar amount of space continues to be available for cars (road space, parking, etc.), and is not reduced to make more space for trees.	Living from
12. Space for housing developments	Space is made available for new housing developments, and the roads, public transport, and other infrastructure needed for them. This could mean a loss of trees and reduced space for new trees.	Living from
13. Local food production	More food consumed in the city is produced locally, directly in and near the city. This includes food linked to trees, such as fruit, nuts and honey.	Living from
14. Tourism	The city is more attractive to tourism.	Living in, living from
15. Nature-based jobs	There are more jobs in the nature sector, such as in maintaining green space, arts, therapeutic activities in nature, and environmental education.	Living from
3. A set of twenty-three concrete policy and community actions, including ten actions relating to the physical landscape, ten social and legal actions, and three actions relating to decision-making and management responsibility (Table S1 in Supplementary Material).
4. A template deliberative process design and facilitation plan for a one-off community workshop of approximately three hours, where participants are asked to consider the visions and then discuss and prioritise the outcomes and actions. The workshop process focuses on building consensus around priorities but allows for minority opinions to also be registered. The collectively prioritised outcomes and actions can be used as the core building blocks for developing a composite synthesis local vision (for an example, see Text S2 in Supplementary Material). The main steps in the workshop process are depicted in Fig. 2.
5. A template pre-workshop questionnaire, where respondents are asked to reflect on broad values and specific values of treescapes, to consider the visions in advance, and express their likes and dislikes, to prompt structured reflection on the visions before the workshop.
6. An open-access online course, documenting the tool and its potential uses, and providing a repository for all the materials, plus supplementary resources on best-practice facilitation^14^.

**Fig. 2:**
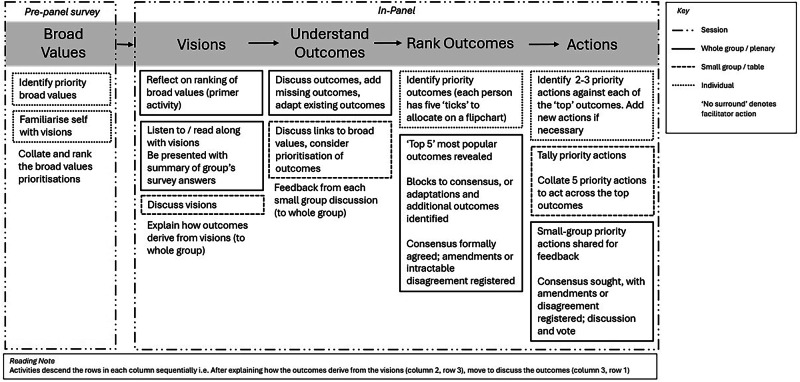
The deliberative process for implementing the tool.

Many of the tool’s outcomes (Table 1) and actions (Table S1 in Supplementary Material) reoccur across the four different visions but are approached differently, and in different combinations. This encourages participants to consider them not just in an abstract way, but within one or more of their original narrative contexts, and so more actively spotlighting different values. The vision narratives are explicitly designed for pluralism and provocation (see Box 1 for living *as*, Box 2 for living *with*, and Supplementary Material for living *in* and *from*). Rather than asking participants to adopt a single preferred frame, they are meant to widen the scope and diversity of values considered, to help expand and deepen thought and discussion about the ways people relate to the natural environment.

Core to Tree Value Visions are concepts of deliberation, relational values, and storytelling as a way to express values. Deliberation is a process by which something can be considered, evaluated or appraised. Group deliberative processes usually involve the following elements^25^: 1) sharing information and learning to form reasoned opinions; 2) expressing reasoned opinions and values as part of civil engagement, respecting differing views, openly expressing disagreement, providing equal participation, and evaluating and re-evaluating positions; 3) identifying, and critically evaluating options or solutions to address problems, considering potential consequences and trade-offs.; and 4) integrating insights from the process to establish specific value priorities, and finding agreement on preferred options through well-informed and reasoned, non-coercive discussion. Such communicative reason refers to forms of rationality that emerges through dialogue oriented toward mutual understanding, rather than rationalities based on enactment of power^26,27^. However, deliberation should not be seen as a rationalist exercise to the exclusion of emotion, embodiment and lived experience^28–30^. The narrative form of Tree Value Visions helps participants engage with these dimensions.

Deliberative processes can have important social justice limitations, depending on implementation. For example, the legitimacy of deliberation depends on inclusive social representation and effective process design and facilitation to manage potential power differentials and to support those with limited prior experience to feel confident^25^. These issues are practically attended to in the facilitation plan and supporting material provided with the tool.

Deliberation has also been considered in the context of institutionalising MSJ. However, this has thus far remained largely conceptual^31^, with some small-scale experiments^32^. A key way to represent non-humans in deliberation is through ‘proxy representation’, i.e., where some deliberative participants are explicit advocates for those interests and justice claims. Proxy representation is compatible with the rapid deliberative process within Tree Value Visions, but not the primary way in which MSJ is operationalised through the tool. Rather than delegating specific individuals as advocates, the innovative nature of Tree Value Visions lies in engaging all participants with non-human and more-than-human perspectives. It does this through the living *with* and living *as* nature visions (Boxs 1–2), which facilitate MSJ in terms of ensuring the consideration of nonanthropocentric, holistic, and relational worldviews^23,33^, and intrinsic and relational values that reflect the interests of trees and treescapes and our bonds with them.

Both living *with* and living *as* treescapes speak to the intrinsic values of trees, but the former considers this through a more dualistic biocentric framing of making space for (and so protecting) other-than-humans. Living *as* takes a more-than-human view of nature, emphasizing trees as community members and proposing actions that express and encourage kinship with trees, such as granting trees legal personhood and social measures to encourage connection to and oneness with nature for both children and adults. In this sense, living *as* bridges the biocentric but less relational living *with* view with the more relational, place-based, weak anthropocentric living *in* treescapes vision. Living *in* treescapes, like living *as*, emphasizes relational values but is geared primarily towards human wellbeing (and relationships) without giving full attention to the intrinsic needs or agency of trees and their associated flora and fauna. Finally, living *from* treescapes expresses a natural-capital-based framing of treescapes as an economic resource.

The four visions each form a story of future treescapes and are grounded in the stories elicited from around 90 residents across three UK cities. Storytelling, as a creative method, focuses on the elicitation of personal stories to form narrative accounts of meaning and value, linking descriptive and normative statements towards an overall unity^34^. Stories often indirectly communicate value judgements of all kinds^35^, providing affirmation of what is important, thus linking to a sense of identity^36^. Narratives express symbolic representations of the environment, reflecting cultural and place identities^37,38^. The telling of stories provides space for relational values to be conceived, confirmed and expressed through the discursive nature of the form. Integration with deliberative democratic approaches that establish common priorities can address key limitations of storytelling as a sociocultural valuation method; stories express what is valuable, but not necessarily which values are most significant or how to resolve competing narratives^35^.

Values include ‘broad values’ (our overarching life goals and principles, e.g., honesty, wealth, fairness, tradition, harmony with nature) and the specific values we ascribe to treescapes which express their importance^10,15^. Relational values are a type of specific values that in this context express the meaningful relationships with treescapes, and among people through treescapes^16^. They express relations that can be deep-rooted and central to place and community, and which lose meaning when reduced to a merely instrumental understanding. They thus contrast with instrumental values (signifying the importance of nature as a substitutable means to human ends) and intrinsic values (nature’s inherent worth, independent of people valuing their relationships with it)^5,7,16^.

Relational values have been identified as key constituents to a good life, to individual and collective identities, to understandings of nature as sacred, and the ways that nature supports social bonds between people^7^. Within the stories that underpin Tree Value Visions, examples of relational values include the ways that people can feel at home within treescapes, memories of growing up playing in trees, feeling comforted by the longevity, serenity and steadiness of trees in difficult times, the beauty of treescapes, their role as places to meet with other people, and the uniqueness, cultural significance or heritage of particular trees and stories about them. Reciprocity with nature is often an important element of relational values, reflected in people both benefiting from trees and feeling care both from and for them. Here, trees can be seen as relatively passive contributors, but also as expressing agency as members of more-than-human communities that people live and interact with over the years and thus want to protect. Because relationships often harbour meaning and emotion, their specific values are highly contextual and not straightforward to substitute. This can explain how, for example, even limited tree felling, or changes in access or property rights to treescapes, can attract significant community protest^39^.

Because of relational values’ typically place-based, contextual, non-substitutable nature, and not being widely incorporated into institutionally normalised data generation, they have generally been elicited through primary research. The challenge of eliciting qualitative data and designing participatory processes from scratch in the context of resource-deprived local authorities and other local actors^40,41^ can reinforce the poor integration of relational values in treescape planning and management. An important rationale for Tree Value Visions was to provide a pre-developed, pre-tested and straightforward to apply participatory process, suitable for relatively low-resource application by local treescape stakeholders, that nonetheless supports place-based discussion.

Box 1 Living *as* trees and treescapes – Oneness and harmony with trees “In our city, we started to think more and more about our connection with trees, realising how much we gain from recognising them as a key part of our community. Although they had always been there, we never really ‘saw’ them. Sometimes we treated them as objects, sometimes as an environment to protect, but what we had not really noticed was their aliveness, their beingness, the effect they would have on us when we took a moment to connect with them. Realising the power of this connection, we decided to make trees ‘green citizens’. They already pay their council tax in kind by cleaning the air, providing shade and protecting us from floods. They are active participants in the life of our city, volunteering just like so many of us do when we care for friends and relatives.

We brought together policy makers, tree officers, local businesses, artists, researchers and community groups to develop a long-term vision that supports these values of oneness and living in harmony with nature. First, we wanted to address the objectification of trees – how could we prevent people from just treating them as a thing? Local government policy now stipulates that a tree is planted for every child born or adopted. We also name trees after the children, and we created a register of all the named trees. People who move into the city are also invited to plant a tree or adopt an existing one. We organise weekly planting ceremonies in each ward so that new people and parents can connect with their trees and with other people locally. A few people met the love of their lives that way! Our digital maps allow people to trace family lines across trees and people and see connections with trees in twin cities. There’s a hope that many trees will become historic or heritage trees because they all have a story to tell that can be shared between generations.

Because we recognised that trees pay their tax in kind, we thought there could be no taxation without representation. Citizens are on a register of guardians and receive a short training (a bit like being on jury duty) and they represent their tree whenever it could be affected by a new development. We also made some big changes to expand the treescape. We improved and created small mixed-age community woodlands dotted around the city that felt like they were a natural part of the community and connected them to each other as much as possible. We issued a planning requirement that all new developments and existing streets must be treelined unless there are strong overriding impediments. We set up social enterprises specifically to support planting trees in private spaces. GPs offered more green prescriptions, and we shifted charity funding from indoor to outdoor activities, with many people involved in managing the treescape through volunteer-run ecotherapy activities. Wildlife is doing well, though some ecologists have argued that the treescape is not optimal ecologically, because it is quite distributed and accessible, but with not so much focus on large reserves.

Most important of all, we developed a policy to maximise child engagement with trees through planting and pruning trees for tree climbing, den making, foraging and other sensory activities, embedding forest schools in every primary school and bushcraft in every secondary school curriculum, supporting parents, and ensuring inclusivity for those with additional learning needs.

Overall, this led to quite an organic way in which we met government targets for tree-planting. Though these were originally created to combat climate change, our relationships changed – with the trees, with nature, with each other and with ourselves – and we became healthier physically and mentally through nature connection and being outside, more community focused, and with much happier kids.”

Box 2 Living *with* trees and treescapes - Healthy ecosystems and protecting the environment “In our city, we wanted to make more space for nature, and trees are a key part of that. Biodiversity, trees and the animals and plants that depend on these deserve to be protected for their own sake. They are also important because of their life supporting services - they are an essential part of the healthy ecosystem that we all depend on and need to maintain to adapt to climate change.

Our strategy was twofold, we wanted to create new areas of woodland for nature to thrive, and make sure treescapes were ecologically interlinked throughout the city and surrounding areas. We developed the city into a living landscape, connecting existing green spaces and adding trees and hedgerows strategically to provide places for wildlife to live and cover through which it can move. Maintaining and extending trees along highways and arterial routes into the city and creating wood meadows near key public places have added shade, urban cooling and cleaner air and brought an abundance of birds, insects and other wildlife. Large new national nature reserves have been created outside the city, with a focus on creating spaces that were large enough to support populations of species that were threatened by extinction or that had disappeared from the area in the past.

Tree planting is focused on maximising ecological benefits, so planning is led by ecological experts supported by citizen scientists. Reviving river and canal sides and expanding hedgerows has increased the city’s flood protection and is a core part of our network of green corridors. Nature bridges connect green spaces across main roads, street corners and derelict spaces have been turned into pocket parks or tiny forests, with information boards on biodiversity that the space provides.

Climate resilience has also been important. We selected species for drought and flood resilience and in light of long-term management. The integration of forest school activities in national curriculums has fostered, in successive generations, more knowledge and appreciation for local treescapes and built the traditions and skills for stewardship.

Local businesses and developers agreed to support the council and take responsibility for trees near them as it formed part of their sustainability strategy, to offset carbon, increase biodiversity and support employee wellbeing by providing green views. Residents are actively encouraged by the council and charities to plant trees in and maintain bee, insect and wildlife friendly gardens, back alleys and allotments.

The connected, living landscape has been built with nature as a hard constraint. In other words, developments cannot go ahead if they have a significant negative effect on biodiversity or rare species. This has meant we have had to be very selective in terms of where we’ve been able to expand housing and other development, restricting it to brown field sites and low-grade agricultural land. Because we feel giving space to nature is at least as important as development, developments have become more efficient; we are seeing more compact housing and apartments to make the best use of available space. This does mean that large houses come at a premium.

The green corridor network has also meant less cars in the city. A number of key routes in the centre and connecting suburbs have become safe green lanes for walking and cycling only. This has encouraged more cycling and walking. The constraints on cars have also boosted public transport use and use of cargo bikes.

Overall, we are living much more with nature, wildlife populations have massively increased in the city, and the city is cleaner and more resilient to flooding and heat waves.”

### Development of the tool

The visions were developed through workshops with residents in each of three UK cities with distinctly different treescapes and sociocultural geographies: Cardiff, Milton Keynes and York. Cardiff is a multicultural national capital and post-industrial port city, contending with continued post-industrial redevelopment and a legacy of important tree collections in city parks. Milton Keynes is a post-1960s new town, formerly branded as the ‘City of Trees’, an example of modernist planning approaches following World War II, and at present expanding rapidly. York has large areas of common land and ambitious tree-planting targets, but built heritage (from Roman to contemporary) often competes with trees for prominence in the cityscape. All three cities have declared a Climate Emergency and expect tree planting to play a significant role in climate mitigation and adaptation.

The values elicited and visions developed from the stories in Cardiff, Milton Keynes, and York were subsequently ‘ported’ to two other cities, Camden and Edinburgh. Camden is an inner-city borough of London with a large proportion of transient residents. Relatively few households have access to private greenspace, amplifying the importance of local public treescapes. Edinburgh treescapes are characterised by multiple parks of national historic importance and residents-only communal gardens; set amidst a fast-growing population and economy, with greenfield and brownfield development.

The purpose of the porting was to ‘test’ if the visions, the associated sets of values, and the policy actions and outcomes, were deemed relevant by citizens and potential end users of the tool outside of the original contexts that they were generated in (the places of Cardiff, Milton Keynes, and York). This process was grounded in the notion of ‘horizontal portability’ proposed by Himes et al.^42^ to describe the potential for transferring interpretive evidence of place-based relational values for use in decisions. Horizontal portability seeks to maintain the contextual rootedness of place-based local value expressions, whilst also communicating them across different communities. This allows lessons to be learned and practices to be developed in new contexts, based on ported values. Tree Value Visions seeks to achieve horizontal porting by embedding relational values (alongside other values) in rich visions of the future that were developed from stories elicited in the original contexts. By including a form of rapid deliberation, Tree Value Visions seeks to balance a ‘resource-light’ approach with the need to validate and contextualise ‘ported’ values in new situations.

In each of the three primary research cities, ‘citizen panels’ were assembled with ~30 residents who met three times each. The panels were constructed as mini-demos^43^, representative across gender, age, social class, and ethnicity, and including those with and without pre-existing interests in trees. The specific individuals participating were identified and assembled by a social research panel recruitment agency. Participants were provided with a substantial incentive fee to help ensure representation. In the first workshops, participants were prompted with a recording of a professional storyteller relating tree stories of the past. They were then engaged in storytelling sessions focused on stories of their own relationships with the treescape of their city in the present. Participants were also provided with prompts around the four Life Frames, inviting participants to consider their diverse values and relations with trees and how these were reflected in the stories that they told.

The second workshop developed these same themes in relation to the future, first by telling stories prompted by thinking about the future treescape in a human generational timescale (25 years) or a tree timescale (100 years), or somewhere in between. These stories elaborated on changes in the treescape, how they would come to pass, who would be involved, and what their impact would be. Future storytelling was made more specific by inviting residents to annotate (with text and drawing) self-selected Google Streetview prints on how they would see changes in the treescape. Storytelling was not constrained to desirable futures only, and the results included positive, dystopian and broadly imaginative stories.

Participants’ stories and commentary were qualitatively coded to the four Life Frames on the basis of their definition and characterisation by IPBES^6^, and to different policy-relevant themes (e.g., biodiversity, flooding, transport); codes were validated by a second researcher and where there were conflicting interpretations discussed with a third (in all cases consensus was reached). In parallel, researchers also inventorised treescape-related local and national policy objectives. All of the story elements that constituted outcomes or actions were compiled into a list, cross-referenced against the frames, and, where possible, linked to the policy objectives. The actions were classified from the data as either social or physical actions, and the outcomes as primarily related to community, or nature and environment, or transport, economy and development.

Working with the coded results, the policy inventory, and list of outcomes and actions, individual researchers (AD, HW, JA, JM, JOK) undertook creative writing sessions to draft four distinctive visions that each consciously emphasised a particular Life Frame. Elements of the original stories were chosen to represent the different outcomes and actions and overall framing, where possible using the participants’ own words, for inclusion into a coherent narrative. These were then peer-edited, now considering them as a set (living *as, in, with* and *from*) to ensure clear, distinctive contrasts between them. Finally, supported by further online deliberation, the research team reviewed the visions against the outcomes and actions, which refined the narratives to ensure each vision expressed different sets and that overlapping outcomes and actions were articulated differently according to the visions’ life frames. It is important to note that this was an interpretive-creative process and, while we followed a structured and data-grounded approach, the methodology was by its nature subjective. The aim here was not to attempt complete objective representations of the entire scope of participants' contributions, but to create new synthetic visionary narratives that were in and of themselves authentic artistic creations of the authors, yet also did justice to the creativity of the participants, reflected the essential value dimensions found across the stories and Life Frames, were able to provoke discussion, and were policy relevant.

The third round of citizen panel workshops in the primary research were subsequently designed to evaluate the visions (both their specifics and underlying frames) and to prioritize actions and outcomes to create a single, locally specific synthesis vision for each city, reflecting local priorities, but still rooted in the future stories told in the previous set of workshops. In these workshops, participants were not explicitly asked to consider MSJ as a concept, aside from the elements expressing MSJ within the original visions. However, participants readily recognised that humans were the primary focus of the living *from* and *in* visions and non-humans of living *with*. In living *as*, people and treescapes were seen as inseparable, with this vision focusing on multispecies co-benefits.

The Tree Value Visions tool was subsequently designed to be operable in a single workshop for wider use, based on a concatenated process (Fig. 2). The pre-workshop questionnaire (see Wood et al.^14^) comprises a brief series of questions based on a well-established instrument ^44^ to elicit participants’ broad, overarching values, and an outline of the four visions, asking for short initial responses. This serves to familiarise participants with the visions and prompt active engagement with the frames in advance of the in-person session. The questionnaire was based on questions that had previously been found effective in the original series of panels in Cardiff, Milton Keynes and York. The draft tool was then tested and refined following single, smaller (n = 16 participants) but similarly recruited and representative citizen panel workshops in Camden and Edinburgh. In particular, we considered whether the visions were readily understandable and applicable to the new cities, whether they were able to stimulate rich discussion, and whether the results validated the horizontal porting of values within their narrative contexts through the tool (albeit a limited, one-way ‘port’ rather than a bidirectional communication of values, cf.^42^). Participants themselves were also asked for feedback on whether any elements of the tool did not make sense in their city (they did not identify any issues).

To further validate the tool, we engaged with a panel of potential end-users, e.g., local authority tree and planning officers and members of community groups, environmental NGOs, and environmental consultancies, through an online meeting (*n* = 10). Key representatives of partner organisations (local authorities, Local Government Association, and Forest Research) also attended the citizen workshops as observers. Key feedback points included fleshing out key policy and management challenges at the start of the process, validating the way the visions had represented local treescapes contexts, feedback on the citizen panel workshop process, reflecting on the outputs and their potential uses in different policy contexts, and providing feedback on the draft visions, actions and outcomes, and course background materials. After holding the panels and producing a near-final version of the tool, we held a training session for professional treescape stakeholders, with an open invitation disseminated via professional mass-membership channels. This consisted of delivering a three-hour online training in use of the tool with further potential end-users (n = 15) who had not had any previous role in helping develop it, which led to final more minor refinements to the materials.

### Uses for Tree Value Visions

Tree Value Visions provides end users with a tool to rapidly identify actions that can enhance the value of, and the value derived from, an area’s trees, achieving a rich picture of priority values without the need for elaborate bespoke primary qualitative data gathering. The tool is designed for application in contexts of limited discretionary spending by local authorities. The tool can develop packages of actions for multi-stakeholder implementation, bundled to provide positive-sum relationships and/or resource-efficient cross-sectoral, ‘total place’ approaches^45^, underpinned by shared learning. This addresses the sector context, whereby resource-limitation means tree management is often largely constrained to statutory duties (e.g., health and safety)^40,41^.

Local authorities are, in principle, well aware of the multiple values of treescapes, a point stressed by stakeholders participating in the end-user meetings. This includes various professional expertise relevant to the value of treescapes (e.g., placemaking, greenspace management), and more general awareness of local residents’ diverse values, such as via public consultations or correspondence from residents. However, these fora rarely explicitly attend to the justice claims of non-humans. Furthermore, as discussed above, relational values typically fall outside of the remit of statutory duties. Where urban authorities lack resources for non-statutory spending, the challenge is to provide opportunities for stakeholders to secure and increase relational values as a co-benefit of achieving policy aims linked to statutory obligations in both environmental management and wider policy areas, such as health and social care, transport, housing, and education. Here, Tree Value Visions can be used to identify and build community support for policy and management priorities through prioritising specific outcomes and actions anchored in deliberation of broad and specific values of treescapes. It helps identify opportunities for consensus building, synergies, and trade-offs across sectors. In so doing, it can be used as a pre-consultation tool, or to inform policy development or appraisal, and help to align individual policies with long-term strategic visions.

For example, in Edinburgh, outcome priorities included improved health and wellbeing, resilience to climate change, space for more housing and public transport, community participation and cohesion, and an abundance of natural life in the city. An action package was developed that included a policy for child engagement with nature, green health prescriptions, planting new small community woodlands, a treescape-based business development plan, and balancing expert and community management. Residents expressed strong support for strengthening outdoor education and actively promoting play in treescapes, both because of the relational wellbeing value for children and because this provided a vector for growing a generation of treescape advocates. On the other hand, they were hesitant for trees to be prioritised over housing developments; the same issue prompted debate in the other cities, which sometimes made different choices. The outcomes of such packages of actions can be implemented through existing instruments such as local tree strategies and carbon target-related strategies, but also in, for example, local development plans, local authority educational policies and business improvement strategies.

For elected representatives, the deliberative tool complements representational democracy and ongoing community engagement or casework. Overall, it can be used to increase the range of views, stakeholders and demographics to be engaged with, through a rapid process that can be anchored in voluntary stakeholder participation (e.g., community groups, third sector organisations) and/or representative public sampling, and a structured method for prompting discussion across a holistic range of life frames, outcomes and actions. It provides a low-resource method to identify risk, support, or opposition for different political and policy options linked to treescapes.

For community groups, third sector or private organisations, Tree Value Visions can be used to identify priorities that align with organisational goals. It can gather evidence that broadens the conversation around local treescape management beyond the relatively narrow sets of values and indicators associated with statutory duties. Moreover, Tree Value Visions can be used for building alliances between different interests, e.g., linking environmental management to health and wellbeing. For children and young people, the tool’s everyday language and straightforward methods mean the direct inclusion of youth representatives is possible.

### Tree Value Visions, relational values, and multispecies justice

A key rationale for developing Tree Value Visions was to provide a mechanism for better addressing relational values and MSJ within local treescape decision-making. Relational values are addressed by highlighting meaningful, place-based and reciprocal values within the visions (particularly, but not exclusively, living *in* and *as* treescapes), and by proposing policy measures informed by these values. For example, the living *in* vision imagery shows people socialising and children playing within treescapes, and the text (see Supplementary Material) frames the importance of treescapes as place, home and heritage. MSJ was reflected by articulating the needs and rights of other-than humans and again reflecting these in specific outcomes and actions. For example, the living *as* vision imagery illustrates a mixed urban community of people, trees and wildlife and expressing values of care, harmony and oneness, while the text proposes de-objectifying trees and policies such as tree citizenship rights and legal tree guardianship.

Across the five cities where the tool was initially developed and then tested, participants expressed relational values associated with trees or treescapes with which they had particular connections. They prioritised actions and outcomes based on these relational considerations and on doing justice to trees and associated wildlife. While a full analysis of participants' stories and deliberations is beyond the scope of this paper, the following serves to illustrate how participants responded to the use of Tree Value Visions and helped inform development of the tool in their cities in relation to these two topics.

Relational values were often associated with strong memories or built up over years of repeated contact, or where trees or treescapes were fundamental to learning about nature or oneself. The two most prominent foci for relational values were the importance of treescapes and connecting with trees for mental health (based on meaningful, particular relationships with treescapes rather than merely generalisable instrumental values), and their role in supporting community cohesion (relations with nature supporting relationships among people). Participants highlighted how trees and treescapes are a place or focal point for relief from life’s stresses, emotional grounding, intense emotional growth or healing, which maintains or improves their mental health.

“I lost my husband …But I have a dog, so the dog got me out … I honestly feel like doing that and seeing those trees and being in that environment really saved me; it got me out of a very dark place. And the trees for me are just reassurance … They’re always there. They really put things in perspective …” (York)

“…outside my house it’s just surrounded by trees… Sometimes with mental health issues, every time, morning before my daughter wakes up, I’m outside with a cup of coffee, and I just feel like I can talk to somebody, even if I’m not talking to somebody…” (Milton Keynes).

Where participants were able to enact care, these relationships would become more bi-directional:

“…if you like, the [two cherry trees are] almost an embodiment of the two children and they are looked after almost better than the kids in actual fact. So that’s those two and they’re doing well. And it’s interesting, because the kids have become attached to them because every time then in the spring they come out into blossom, they chart the progress….(York)

Participants also discussed how treescapes, including parks or woodlands, facilitate meeting up with family and friends, and social activities, including communal stewardship of the treescape. In turn, these activities build community cohesion.

“…the problem is it’s not maintained, and the drains are blocked… the council used to… But we have organised in my street a WhatsApp group, and we get out there, and we sweep it. People like that because we have mulled wine, and we have drinks and this and that; so it’s the community coming together and reacting.” (Cardiff)

Differences between city contexts lead to different meanings of social cohesion relating to treescapes. For example, Camden was described as not ‘a community-driven space’, due to its fluctuating population (particularly linked to student housing and rising prices). There was a hope that creating more spaces enabling encounter and social interaction – rather than just lines of trees along otherwise-unchanged streets remaining predominantly used for instrumental transport – could instigate community:

“If you create the green spaces, then people will come and use them, the community will come, people will connect.” (Camden)

Edinburgh participants focused on strengthening existing communities and creating intergenerational ties. Green community spaces would allow different generations and different groups to spend time together and could over time become a constitutive part of identity tied to the local community, itself changed through incorporating these new spaces:

“… this sense of place… something that is not that tangible…Having these community spaces that could well over time become a recognisable part of the community. The different generations could experience - and the different groups, who maybe sometimes do not meet, can spend time together.” (Edinburgh)

Participants regularly mentioned wildlife of all kinds on equal terms in the stories that underpinned the visions of the tool. Here, participants spoke to both the living *with* frame, considering treescapes as a space where nature could co-exist with people, and to the living *as* frame, where trees were considered as community members, where the trees would evolve to facilitate that relationship.

“Now the trees that line this street are full of long-tailed tits, wagtails, parrots, storks, herons and so much more. The canopy is full of life with butterflies, bees and other insects buzzing, adding to the music I hear when I [go] through the market buying my locally grown vegetables.” (York)

“I often feel scared when jogging in the evenings alone. So I planted loads of trees along my usual jogging path. In 25 years, there are lights in the trees (which look like eyes glowing in the dark) that light my way. In 100 years, the trees come alive at night and hug my great-great-grandchildren when they’re scared.” (Milton Keynes)

These different stories illustrate the emphasis by participants on meaningful relationships between people and trees, including relations of place, relations of (sometimes mutual) care, and relations that signify multispecies kinship or multispecies community. Such relations tended to underpin notions of MSJ more often than more abstract philosophical reflections. Stories of the future, from dystopic to utopic and fantastic, spoke most clearly to MSJ. Here, trees could be passive subjects, treated badly or with care, and potentially enfranchised by people, but they were also regularly described as active participants in the life of the city or the agents of their own emancipation.

“I grow in the rain and the sun. I lived from the land and breathe the air. I watched them live. I watched them thrive. I watched them destroy. I watched them die. Some of them tried to help but the damage was done. They destroyed my home and my friends.” (York)

“A dream comes true! In the next century all trees will have a ID card as York citizens. Because they will be considered as living citizens with the same rights such as voting like the human citizens.” (York)

“In the future trees have become sentient and have learned to speak. At first, relationships between people and trees were strained, the trees were angry at how they had been treated over the centuries. But slowly people and trees started to build friendships. Trees told tales of all the history they had seen. People learned how to live differently. The trees and people learned to coexist and thrived together, living in our shared planet in harmony.” (Cardiff)

Attendance to MSJ became more explicit in the prioritisation exercises undertaken by the panels. Within Tree Value Visions, to develop a city-specific vision of the future the panel participants are asked to first prioritise a set of outcomes drawn from the four narrative visions (Table 1), and secondly to identify actions that best achieve the priority outcomes (Supplementary Material, Table S1). To illustrate the degree to which justice claims from non-humans can potentially be satisfied through the tool, we classified the set of actions and outcomes that participants in the five cities prioritised according to whether the outcomes and actions were of primary benefit to people, or to non-humans (e.g. ‘trees have legal guardians’, ‘new developments have biodiversity as a hard constraint’), or of mutual benefit (e.g. ‘tree-lining streets wherever possible’, ‘tree planting focused on drought and flood resilience’).

**Fig. 3:**
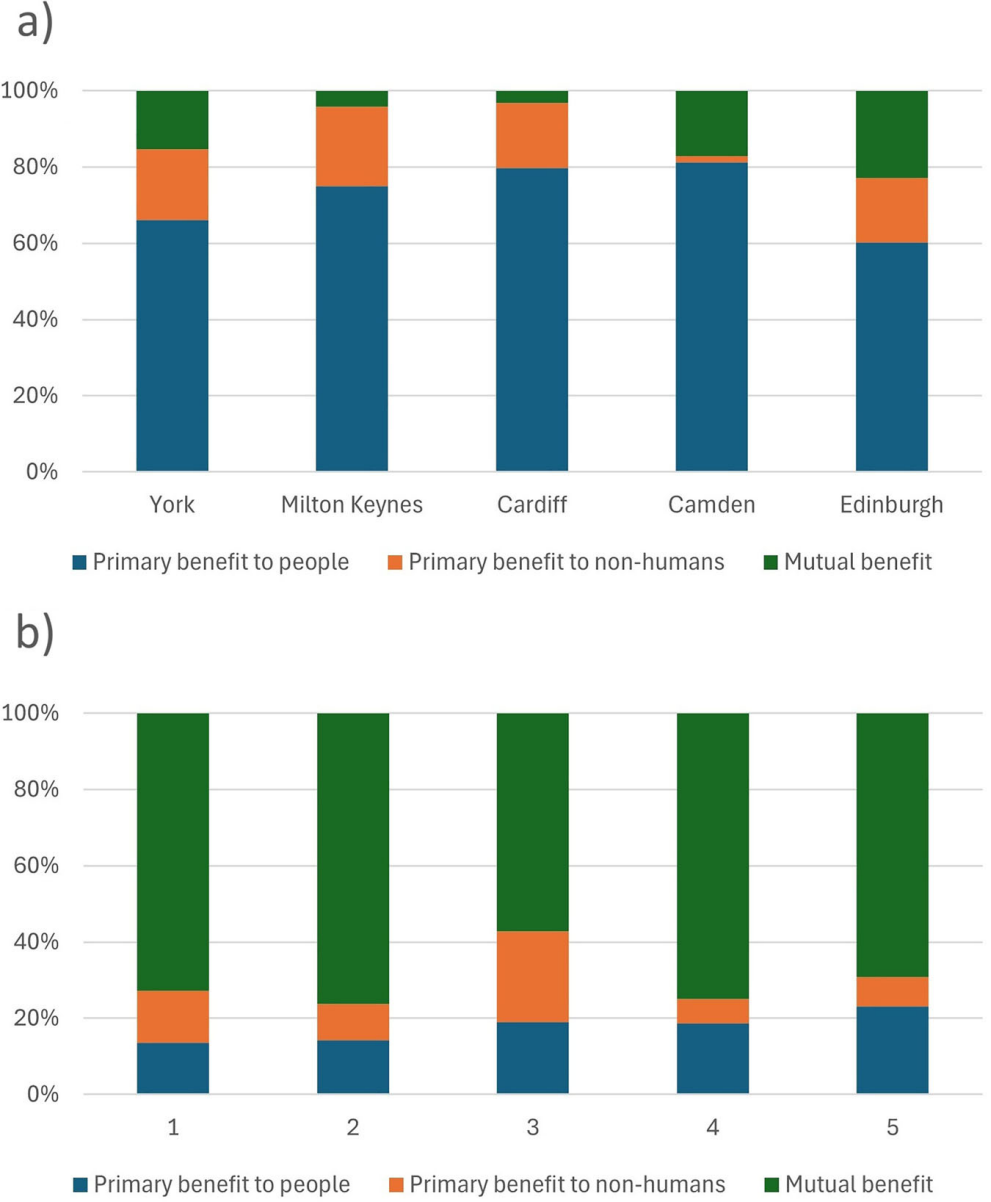
Beneficiaries of citizen panel priority outcomes and actions in the five cities where Tree Value Visions was developed and tested. **a** Outcome beneficiaries. **b** Action beneficiaries.

In terms of outcomes, the panels in all five cities favoured those of primary benefit to people (Fig. 3a). However, when participants prioritised actions (Fig. 3b), all panels chose to prioritise actions with mutual benefit. This suggests that while teleological concerns were predominantly anthropocentric, in prioritising actions they actively sought out synergies between people and nature. This is not uncommon in deliberative processes around the environment where people are asked to take the seat of decision makers^22^, and where communicative action through deliberation may seek to enhance social value by meeting multiple broad values and interests, including those of non-humans.

In some moments of deliberation, the justice claims of non-humans were actively prioritised with full awareness of the trade-offs. For example, in York, following extensive discussion, the vast majority of the panel agreed that biodiversity should be a hard constraint on developments, rather than allowing the possibility of offsetting elsewhere, which people felt was too uncertain and did not do justice to existing nature that would be sacrificed. This was despite the awareness that such constraints would impede housebuilding in the midst of a prominent societal housing shortage.

Participants also reflected somewhat on the importance of integrating more bio- and eco-centric views within future shared social values, where they recognised the importance for future generations to be more closely connected to nature. Policies for engaging children and integrating more nature connection activities into formal education were selected as a priority in all the cities. In this sense, participants appeared to some degree to think ‘polycentrically’^6^, shifting between human justice claims in terms of health and wellbeing and fair access to trees, to the needs of future generations and the justice claims of non-humans, and sometimes more-than-human communities. Discussion also included the potential for a societal shift in the ethical envelope^46^, where future generations would take greater responsibility towards meeting non-human justice claims; congruent with key high-impact leverage points for transformative change towards just sustainability identified by IPBES^10^.

In terms of the different Life Frames, and the degrees and ways they attended to MSJ claims, there was an interesting difference between the three initial cities where the tool was developed based on extensive storytelling and multiple rounds of deliberation, and the two cities where the tool was tested through single citizen panel meetings. In the initial cities, the storytelling and extended process seemed to open the imaginary more towards the living *as* frame of trees as citizens. However, the less creative and shorter process in Camden and Edinburgh more quickly seemed to close off these options through doubts about their practicality, with perceptions of them being more ‘extreme’ compared to the more familiar nature conservation narrative reflected in the living *with* frame, and the social cohesion and human justice appeals of the living *in* frame (emphasizing the rights of people to accessible greenspace). Besides the extent of the process, a further difference was that the panels in the original three cities were almost twice the size of those in Camden and Edinburgh. In Cardiff, York, and Milton Keynes, the larger panels meant that there appeared to be more individuals who from the start gravitated towards the more radical elements of the visions and informally could be seen to actively proxy-represent nature. The greater mix of participants and longer process thus helped to normalise ideas such as the agency of non-humans more so in the original panels than in the latter ones.

Another barrier was participants’ felt community capacity to achieve MSJ through some of the bottom-up actions proposed, such as a proposal to plant a tree for every child born, and that trees should be represented by community guardians in decision making. Shifting from more conventional, top-down approaches to nature conservation, and towards embedding trees within communities through extensive community tree planting, self-managing green spaces, and representing trees in formal processes, participants frequently felt this too great a practical burden, particularly against the current backdrop of social deprivation, cost-of-living, and time-stretched households. As such, measures attending to social justice, including and beyond treescapes, could support communities in feeling more able to take more responsibility for MSJ.

## Conclusions

Tree Value Visions provides an ‘off the shelf’ process for decision-makers designed to better integrate diverse values (and specifically relational values) into local treescape management, whilst integrating specific sets of outcomes and actions that can serve to meet the justice claims of humans and non-humans. Within the process, participants are encouraged to engage with different ways of thinking about their relationship to trees and the treescape, and the values they prioritise in those relations, including prosperity, place, healthy ecosystems, or oneness and harmony with nature.

By providing a set of rich narratives, relational values are not abstracted but horizontally ported^42^ within the context of their original narrative elements. Through this process, relational values retain place-based local value expressions, rooted in context, whilst being ported into different communities, who are invited to reflect on the relevance of these stories to their own experiences, contexts, and applications.

In practice, use of the tool can encourage people to think about synergetic solutions for people and nature, providing a practical way to deliver evidence around nature-based solutions to support ‘total place’ approaches in local governance^45^, and creative approaches to understanding how citizens interact with and make sense of the public realm^46^. Within this, elicitation of relational values that connect care for and from trees to social bonds with other people, place, health and wellbeing provides a ‘glue’ for linking social and environmental aspirations^47^.

Across the five cities, the tool enabled participants to articulate and attend to the needs of other species. However, this happened more strongly within the larger panels and longer process of the original cities. Where people harbour predominantly anthropocentric worldviews, it may be difficult to give full consideration of MSJ without more extensive deliberation and by including participants who can explicitly advocate on behalf of nonanthropocentric, holistic and relational views, such as those expressed in the living *as* trees vision – either through larger panel sizes or by explicitly inviting people who can represent such views and non-human justice claims.

However, tools such as this are by necessity a compromise between the realities of practical and financial resources available to decision makers and other actors that might want to use such tools, and different theoretical ideals and conditions of such processes regarding deliberative democracy, value pluralism, procedural justice and interpretive and creative epistemologies. It is a matter of judgement as to whether they are ‘good enough’ to achieve their aims in terms of greater participation, value pluralism, and representation of MSJ claims, and would improve decisions in terms of quality, inclusion and legitimacy versus if they had not been used, and whether these improvements are achieved efficiently. While more extensive deliberation with greater integration of creative methods could improve the degree to which participants are able to broaden their imagination of future possibilities^48^, in the cities where the shorter process was used, participants were still able to recognise and include non-human justice claims in their overall priorities.

What is the most appropriate approach will depend on the potential specific needs to broaden the imaginary and the resources, time and capacity available within any decision-making context. Where there are specific, highly contested or high-stakes environmental justice issues at play, a more elaborate and creative deliberative process is likely to be beneficial, potentially coupled with more explicit proxy-representation of nature. Where these issues are of less concern, and where it is more difficult to find resources for elaborate participatory processes, the rapid, low-cost template can still effectively support better integration of more pluralistic and relational values and a meaningful degree of emancipation of non-human interests within decision-making processes.

Tree Value Visions is thus provided as an expandable template, and future iterations could include further ‘add-ons’ to the process to enable this deeper deliberation and integration with further creative methods. Other add-ons may consider deeper engagement with other social priorities, such as mental health and wellbeing. Linking multispecies justice to such key social concerns through relationally oriented tools like Tree Value Visions can provide a key lever for building social support for including more-than-human justice demands in decisions.

## Supplementary information

Supplementary information

## Data Availability

Data for the Branching Out project is available from https://reshare.ukdataservice.ac.uk/857960/.

## References

[1] IPCC. *IPCC Special Report On Climate Change, Desertification, Land Degradation, Sustainable Land Management, Food Security, And Greenhouse Gas Fluxes In Terrestrial Ecosystems*. https://www.ipcc.ch/site/assets/uploads/2019/08/Fullreport-1.pdf. (IPCC, 2019).

[2] Climate Change Committee. *Land Use: Policies for a Net Zero UK* (Climate Change Committee, 2020).

[3] United Nations. *General Assembly Resolution A/RES/70/1: Transforming Our World, the 2030 Agenda for Sustainable Development*. https://sdgs.un.org/2030agenda (2015).

[4] O’Brien, L. et al. Exploring the social and cultural values of trees and woodlands in England: A new composite measure. *People Nat.* **6**, 1334–1354 (2024).

[5] Raymond, C. M. et al. An inclusive typology of values for navigating transformations towards a just and sustainable future. *Curr. Opin. Environ. Sustain.* **64**, 101301 (2023).

[6] Anderson, C.B. et al. *Chapter 2: Conceptualizing the diverse values of nature and their contributions to people in The Methodological Assessment Report on The Diverse Values and Valuation of Nature of the Intergovernmental Science-Policy Platform on Biodiversity and Ecosystem Services* (eds. Balvanera, P. et al.) 31–122 10.5281/zenodo.6493134 (IPBES Secretariat, 2022).

[7] Himes, A. et al. Why nature matters: a systematic review of intrinsic, instrumental, and relational values. *BioScience* **74**, 25–43 (2024). 38313563 10.1093/biosci/biad109 PMC10831222

[8] Davies, H. et al. *Delivery of Ecosystem Services By Urban Forests* (Forestry Commission, 2017).

[9] O’Brien, L. Engaging with and shaping nature: a nature-based intervention for those with mental health and behavioural problems at the Westonbirt Arboretum in England. *Int. J. Environ. Res. public health* **15**, 2214 (2018). 30309039 10.3390/ijerph15102214 PMC6210670

[10] Pascual, U. et al. Diverse values of nature for sustainability. *Nature* **620**, 813–823, 10.1038/s41586-023-06406-9 (2023). 37558877 10.1038/s41586-023-06406-9 PMC10447232

[11] Kenter, J. O. et al. Toward a relational biodiversity economics: Embedding plural values for sustainability transformation. *Proc. Natl. Acad. Sci.* **122**, e2314586122 (2025). p. 41004232 10.1073/pnas.2314586122 PMC12519078

[12] Raymond, C.M. et al. Applying multispecies justice in nature-based solutions and urban sustainability planning: Tensions and prospects. *npj Urban Sustain*. **5**, 10.1038/s42949-025-00191-2 (2025).

[13] Chao, S. & Celermajer, D. Introduction: multispecies Justice. *Cult. Politics.* **19**, 1–17, 10.1215/17432197-10232431 (2023).

[14] Wood, P.R.H., Dyke, A., Kenter, J.O., & Zimmermann, A., Tree Value Visions: training course. *OpenLearnCreate*, https://www.open.edu/openlearncreate/course/view.php?id=15149. (2026).

[15] O’Neill, J. The varieties of intrinsic value. *Monist* **75**, 119–137 (1992).

[16] O’Connor, S. & Kenter, J. O. Making intrinsic values work; integrating intrinsic values of the more-than-human world through the Life Framework of Values. *Sustain Sci.* **14**, 1247–1265 (2019).

[17] Nussbaum, M.C. *Frontiers of Justice: Disability, Nationality, Species Membership*. 10.2307/j.ctv1c7zftw (Harvard University Press, 2006).

[18] Schlosberg, D. *Defining Environmental Justice: Theories, Movements, and Nature*. 10.1093/acprof:oso/9780199286294.001.0001 (Oxford University Press, 2007)

[19] West, S. et al. Relational approaches to sustainability transformations: walking together in a world of many worlds. *Ecosyst. People* **20**, 2370539 (2024).

[20] Hourdequin, M. & Wong, D. B. A relational approach to environmental ethics. *J. Chin. Philos.* **32**, 19–33 (2005).

[21] Jax, K. et al. Caring for nature matters: a relational approach for understanding nature’s contributions to human well-being. *Curr. Opin. Environ. Sustain.* **35**, 22–29 (2018).

[22] Chan, K. M. A. et al. Opinion: Why protect nature? Rethinking values and the environment. *P Natl. Acad. Sci. USA* **113**, 1462–1465 (2016). 10.1073/pnas.1525002113 PMC4760809 26862158

[23] Kenter, J. O. & O’Connor, S. The Life Framework of Values and living as nature; towards a full recognition of holistic and relational ontologies. *Sustain. Sci.* **17**, 2529–2542 (2022).

[24] Kenter, J. O. et al. Loving the mess: navigating diversity and conflict in social values for sustainability. *Sustain Sci.* **14**, 1439–1461 (2019).

[25] Kenter, J. O., Reed, M. S. & Fazey, I. The deliberative value formation model. *Ecosyst. Serv.* **21**, 194–207, 10.1016/j.ecoser.2016.09.015 (2016).

[26] Habermas, J. *The theory of Communicative Action*, Vol. 1. (Polity Press, 1984).

[27] Orchard-Webb, J., Kenter, J. O., Bryce, R. & Church, A. Deliberative democratic monetary valuation to implement the ecosystem approach. *Ecosyst. Serv.* **21**, 308–318.t (2016).

[28] Zimmermann, A., Kenter, J.O. & Dyke, A., ‘The Way Enthusiasm Builds’: frame amplification and emotional reinforcement in participatory policymaking. *J. Bus. Ethics* 1-21 10.1007/s10551-025-06045-2 (2025).

[29] Zimmermann, A., Kenter, J. O. & Wood, P. Values, emotions and views of the future: democratic deliberation as pathway to inner transformation. *Sustain Sci.*10.1007/s11625-025-01778-5 (2026).

[30] Mendonça, R. F., Ercan, S. A. & Asenbaum, H. More than words: a multidimensional approach to deliberative democracy. *Political Stud.* **70**, 153–172 (2022).

[31] Celermajer, D. et al. Multispecies justice: theories, challenges, and a research agenda for environmental politics. *Environ. Politics* **30**, 119–140 (2021).

[32] Pineda-Pinto et al. Realizing multispecies justice through a capability approach to promote nature-based solutions. *npj Urban Sustain.* **5**, 31 (2015). 10.1038/s42949-025-00205-z PMC12119368 40453562

[33] Murali et al. Navigating diverse human–nature worldviews for more inclusive conservation. *Conserv. Biol.* **40**, e70144. (2025) 10.1111/cobi.70144 40937846

[34] O’Neill, J., Holland, A. & Light, A. *Environmental Values* (Routledge, 2008).

[35] McShane, K. Some challenges for narrative accounts of value. *Ethics Environ.* **17**, 45–69 (2012).

[36] Shnabel, N. et al. Demystifying values-affirmation interventions writing about social belonging is a key to buffering against identity threat. *Pers. Soc. Psychol. Bull.* **39**, 663–676 (2013). 23478675 10.1177/0146167213480816

[37] Kenter, J. O. et al. The impact of information, value-deliberation and group-based decision-making on values for ecosystem services: Integrating deliberative monetary valuation and storytelling. *Ecosyst. Serv.* **21**, 270–290 (2016).

[38] Kenter, J. O. et al. What are shared and social values of ecosystems. *Ecol. Econ.* **111**, 86–99 (2015).

[39] Kenter et al. *Shared, Plural and Cultural Values of Ecosystems: UK National Ecosystem Assessment Follow-on Work Package Report 6*. 10.13140/RG.2.1.1275.6565 (2014)

[40] Smith, A., Whitten, M. & Ernwein, M. De-municipalisation? Legacies of austerity for England’s urban parks. *Geogr. J.* **191**, e12518 (2025). p.

[41] Pill, M. Rethinking the ‘local state’ and local capacity. *Local Gov. Stud.* **50**, 998–1007 (2024).

[42] Himes et al. Horizontal portability: a proposal for representing place-based relational values in research and policy. *People Nat.* **7**, 752–764 (2025).

[43] Grӧnlund, K., Bӓchtiger, A. & Setälä, M. *Deliberative Mini-Publics: Involving Citizens in the Democratic Process* (ECPR Press, 2015).

[44] Stern, P. C., Dietz, T. & Guagnano, G. A. A Brief inventory of values. *Educ. Psychol. Meas.* **58**, 984 (1998).

[45] Leadership Centre for Local Government. *Places, People and Politics: Learning to do Things Differently*. 1–64 https://www.leadershipcentre.org.uk/wp-content/uploads/2016/12/LCLG_Learning-History_art_lres.pdf (2010).

[46] Latham, A. & Wood, P. R. Inhabiting infrastructure: exploring the interactional spaces of urban cycling. *Environ. Plan. A* **47**, 300–319 (2015).

[47] Everard, M., Reed, M. S. & Kenter, J. O. The ripple effect: institutionalising pro-environmental values to shift societal norms and behaviours. *Ecosyst. Serv.* **21**, 230–240 (2016).

[48] Edwards, D. M., Collins, T. M. & Goto, R. An arts-led dialogue to elicit shared, plural and cultural values of ecosystems. *Ecosyst. Serv.* **21**, 319–328 (2016).

