# Supplementary material for: Tree Value Visions – integrating relational values and multispecies justice in urban treescape management
Source: NPJ Urban Sustain. 2026 Jul 13;6(1):93. doi: 10.1038/s42949-026-00410-4 (PMC13364657; doi:10.1038/s42949-026-00410-4)
Supplement: Supplementary file 1 — Supplementary information [file 42949_2026_410_MOESM1_ESM.pdf]

Tree Value Visions – Integrating relational values and multispecies justice in urban treescape management: Supplementary information

Text S1: the four Tree Value Visions

Note: The *living as* and *living with* visions are identical to Box 1 and 2 in the manuscript text.

Living *in* treescapes – Trees contributing to desirable places to live

In our city, treescapes and trees increasingly became recognised as a defining feature of the places where we live and work and where we spend time with our family and friends. Trees make us feel at home.

Local residents are meeting to celebrate 25 years since they planted a community orchard together. A child plays with his friends among the blossoms. The orchard, which the child's mother remembers planting when she was a teenager as part of an educational program, now produces fruit that is shared as part of the celebrations. Trees planted by the child's grandfather in a nearby garden are tall enough to provide shade and can be seen from many residents' houses. A treehouse has been built in one of the pine trees. The grandfather smiles up at the treehouse, remembering how much joy it brought his daughter, and now his grandson.

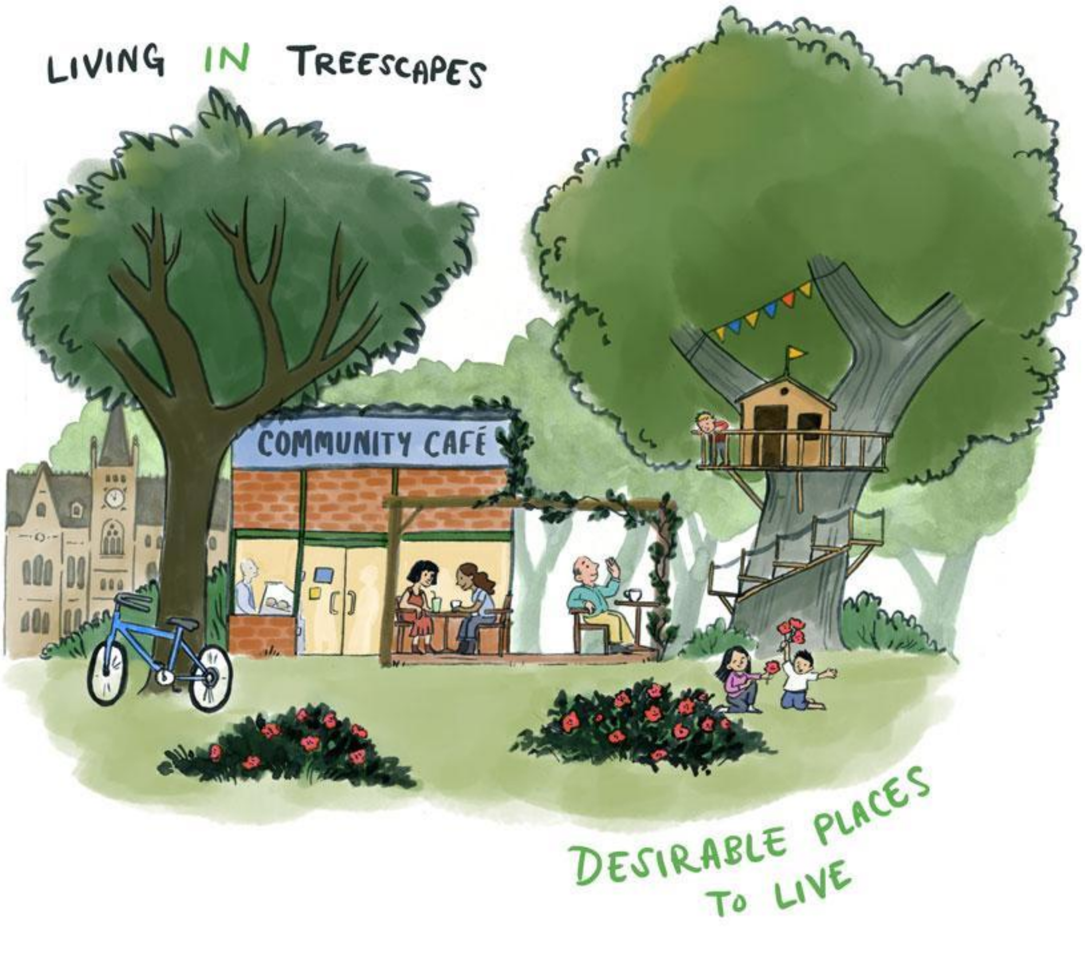

Due to new local government policy, everyone in our city now has access to a community woodland or park that is within 200m of their home. Thanks to these targets, the city is greener than it was when the grandfather was young and worked in the city centre. The paved square he used to walk across to get to his office is now a meadow fringed with trees and colourful flowers.

We focused on tree planting to create green spaces for everyone, for recreation, to meet up with each other, and to benefit our mental and physical health, escaping the stresses of life and the heat waves that we get more often now due to climate change. The inequality that used to be there, where some people would struggle to find accessible

green space, is gone. Green prescriptions are strongly embedded in the health service, and there are policies in place that ensure that hospitals and care homes look out on green space.

We also created a network of green paths throughout the city and put a programme in place to line streets with trees wherever possible. New utilities and broadband cables have gradually shifted to sit under roads rather than pavements to create space for street trees, which does mean that traffic flow is interrupted more often. The rooftops have also become homes for trees, creating new greenspaces in the city centre for people to use and get away from noise and bustle of the pedestrianised streets below.

We developed culture and business plans to take advantage of green spaces, and we have many more cafes, restaurants, outdoor gyms, music and cultural events outdoors, which local people and tourists enjoy alike. Cultural heritage is also important, we've strongly protected veteran trees and old treelined lanes because they are so important to our sense of place, and started to think about which trees and lanes might become heritage for future generations.

Many of the smaller green spaces and community woodlands have devolved management through neighbourhood groups, though supported by the council's tree officers. The tree species planted are prioritised by local people, leading to a diversity of native and non-native species. Many communities choose species that provide colours in the autumn and blossom in the spring. This does mean the treescape is diffuse and focused on amenity, which is not always optimal for biodiversity conservation.

An old willow tree leans over one of the streams providing dappled shade over the water. The willow has seen the landscape change from fields, to town, to urban forest; but tree planting means that this tree is now better connected to the wider treescape. From the laughter and chatter of people enjoying its shade and playing in its branches you can hear how it is providing a home to the people of the city too.

Living *from* treescapes - Prosperity from trees

My company sent me on a mission to learn about the city and its green investment opportunities. As I drove into town, the roads leading from the centre were lined with blossom trees giving a welcoming feeling. From the top of tour buses, tourists took photos of the blossom. Trees make our city attractive to visit, and greening of the city has benefited the tourism industry. Continuing into town, I parked near the city council offices and went in to meet my contact, the green investment manager. They took me up to the roof garden, with a fantastic view over the city.

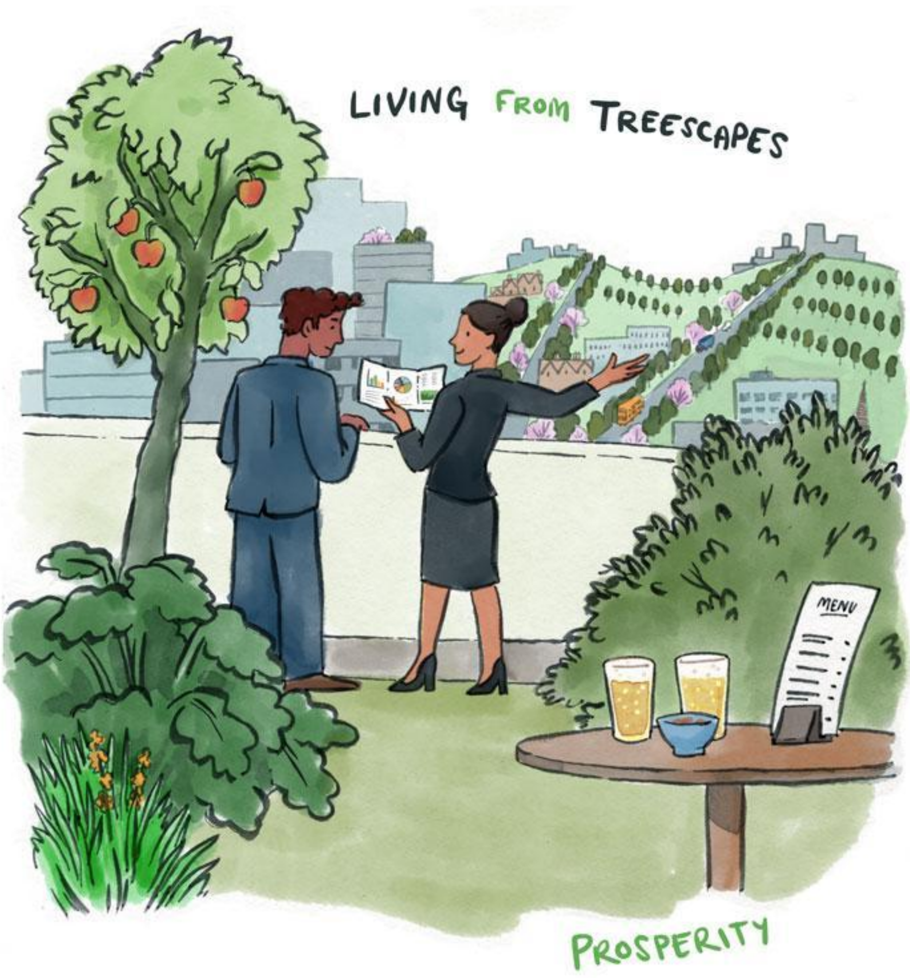

The investment manager handed me a brochure. There were opportunities all over the city. In the centre, exclusive roof gardens for paying customers, with a great looking return. The investment manager pointed out green areas on the edges of the city where there are partnership opportunities to invest in agroforestry, where fruit trees and timber trees are growing amidst other crops and livestock. Tree species that make a mess on the street are now grown in these areas instead and there is less cleaning up for the local authority to do.

The investment manager proudly showed me the radial greenways that stretch from the town centre out to outlying villages and new housing estates. I could see the hedges, full of fruit trees with runners, cyclists and walkers moving alongside them, all saving money on healthcare! Looking up the river, it is possible to make out new areas of trees in the distance. These are planted for several purposes: they are fast growing, to offset the carbon emissions that the city makes, but they also absorb water and slow down the speed by which rain ends up in streams and rivers. The risk of flooding in the city has gone down significantly.

When I went out to eat that evening, the restaurant seemed slightly short of staff. The owner told me that the local teenagers like to get jobs in the agroforestry farms in the summer, and some stay on to learn to do the more skilled jobs like pruning or maintaining the rooftop gardens.

Planning is not primarily managed to protect nature and wildlife for its own sake, but to make use of nature's benefits most efficiently. Woodlands are expanded in some places to compensate for losses in other places that are economically most attractive for development. My company has an

opportunity to build an industrial complex in another city, but we acknowledge that there will be some damage to nature, including an area of trees. If we invest in biodiversity credits in an agroforest here, we can offset the trees and nature we will lose elsewhere and we'll get a nice return on the fruit and other agroforestry products. This will also help compensate for the loss of some old tree-lined lanes just outside of the city that had to make way for new transport links. I'm looking forward to reporting back to the directors next week.

Living *as* trees and treescapes – Oneness and harmony with trees

In our city, we started to think more and more about our connection with trees, realising how much we gain from recognising them as a key part of our community. Although they had always been there, we never really 'saw' them. Sometimes we treated them as objects, sometimes as an environment to protect, but what we had not really noticed was their aliveness, their beingness, the effect they would have on us when we took a moment to connect with them. Realising the power of this connection, we decided to make trees 'green citizens'. They already pay their council tax in kind by cleaning the air, providing shade and protecting us from floods. They are active participants in the life of our city, volunteering just like so many of us do when we care for friends and relatives.

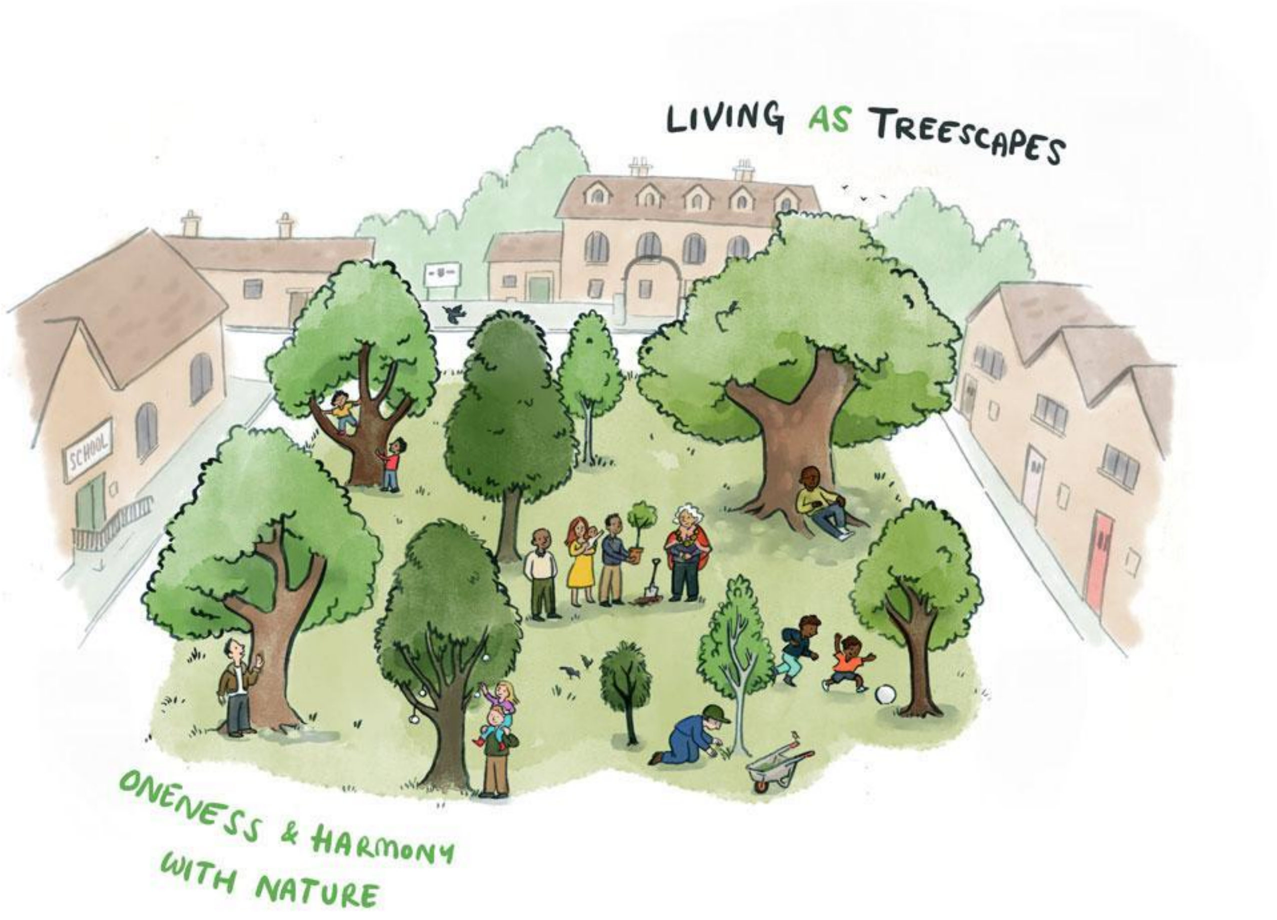

We brought together policy makers, tree officers, local businesses, artists, researchers and community groups to develop a long-term vision that supports these values of oneness and living in harmony with nature. First, we wanted to address the objectification of trees – how could we prevent people from just treating them as a thing? Local government policy now stipulates that a tree is planted for every child born or adopted. We also name trees after the children, and we created a register of all the named trees. People who move into the city are also invited to plant a tree or adopt an existing one. We organise weekly planting ceremonies in each ward so that new people and parents can connect with their trees and with other people locally. A few people met the love of their lives that way! Our digital maps allow people to trace family lines across trees and people and see connections with trees in twin cities. There's a hope that many trees will become historic or heritage trees because they all have a story to tell that can be shared between generations.

Because we recognised that trees pay their tax in kind, we thought there could be no taxation without representation. Citizens are on a register of guardians and receive a short training (a bit like being on jury duty) and they represent their tree whenever it could be affected by a new development. We also made some big changes to expand the treescape. We improved and created small mixed-age community woodlands dotted around the city that

felt like they were a natural part of the community and connected them to each other as much as possible. We issued a planning requirement that all new developments and existing streets must be treelined unless there are strong overriding impediments. We set up social enterprises specifically to support planting trees in private spaces. GPs offered more green prescriptions and we shifted charity funding from indoor to outdoor activities, with many people involved in managing the treescape through volunteer-run ecotherapy activities. Wildlife is doing well, though some ecologists have argued that the treescape is not optimal ecologically, because it is quite distributed and accessible but with not so much focus on large reserves.

Most important of all, we developed a policy to maximise child engagement with trees through planting and pruning trees for tree climbing, den making, foraging and other sensory activities, embedding forest schools in every primary school and bushcraft in every secondary school curriculum, supporting parents, and ensuring inclusivity for those with additional learning needs.

Overall, this led to quite an organic way in which we met government targets for tree-planting. Though these were originally created to combat climate change, our relationships changed – with the trees, with nature, with each other and with ourselves – and we became healthier physically and mentally through nature connection and being outside, more community focused, and with much happier kids.

Living *with* trees and treescapes - Healthy ecosystems and protecting the environment

In our city, we wanted to make more space for nature, and trees are a key part of that. Biodiversity, trees and the animals and plants that depend on these deserve to be protected for their own sake. They are also important because of their life supporting services - they are an essential part of the healthy ecosystem that we all depend on and need to maintain to adapt to climate change.

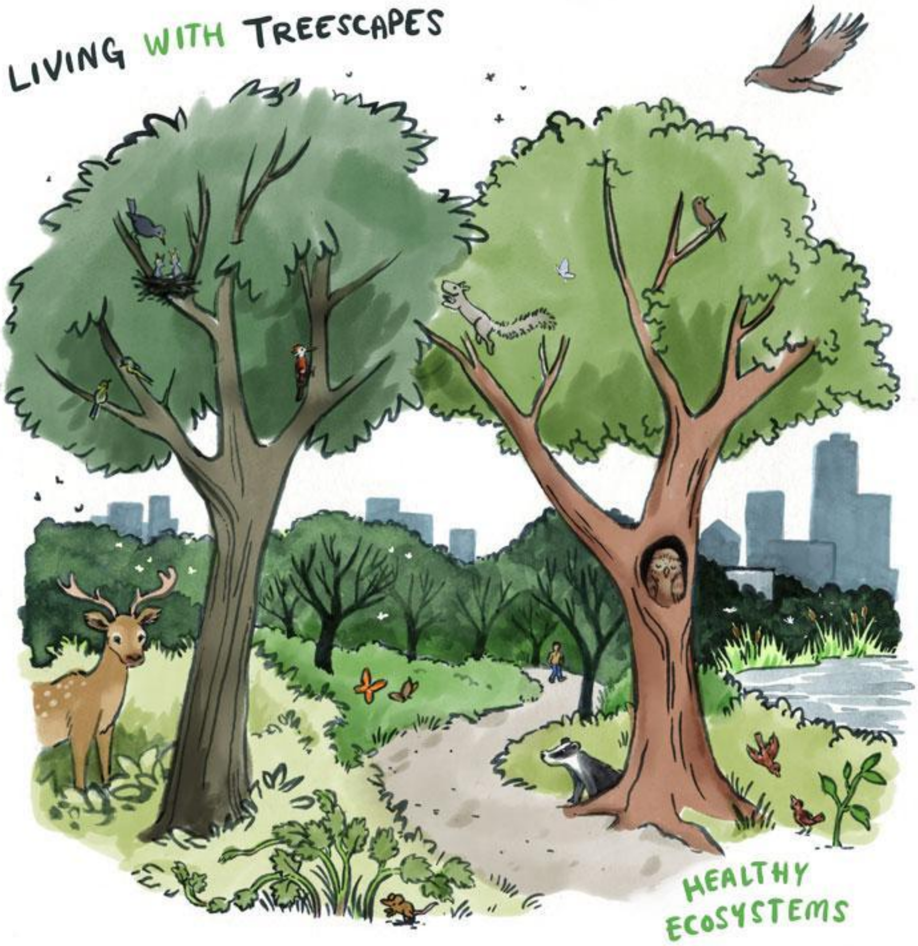

Our strategy was twofold, we wanted to create new areas of woodland for nature to thrive, and make sure treescapes were ecologically interlinked throughout the city and surrounding areas. We developed the city into a living landscape, connecting existing green spaces and adding trees and hedgerows strategically to provide places for wildlife to live and cover through which it can move. Maintaining and extending trees along highways and arterial routes into the city and creating wood meadows near key public places have added shade, urban cooling and cleaner air and brought an abundance of birds, insects and other wildlife. Large new national nature reserves have been created outside the city, with a focus on creating spaces that were large enough to support populations of species that were threatened by extinction or that had disappeared from the area in the past.

Tree planting is focused on maximising ecological benefits, so planning is led by ecological experts supported by citizen scientists. Reviving river and canal sides and expanding hedgerows has increased the city's flood protection and is a core part of our network of green corridors. Nature bridges connect green spaces across main roads, street corners and derelict spaces have been turned into pocket parks or tiny forests, with information boards on biodiversity that the space provides.

Climate resilience has also been important. We selected species for drought and flood resilience and in light of long-term management. The integration of forest school activities in national curriculums has fostered, in successive generations, more knowledge and appreciation for local treescapes and built the traditions and skills for stewardship.

Local businesses and developers agreed to support the council and take responsibility for trees near them as it formed part of their sustainability strategy, to offset carbon, increase biodiversity and support employee wellbeing by providing green views. Residents are actively encouraged by the council and charities to plant trees in and maintain bee, insect and wildlife friendly gardens, back alleys and allotments.

The connected, living landscape has been built with nature as a hard constraint. In other words, developments cannot go ahead if they have a significant negative effect on biodiversity or rare species. This has meant we have had to be very selective in terms of where we've been able to expand housing and other development, restricting it to brown field sites and low-grade agricultural land. Because we feel giving space to nature is at least as important as development, developments have become more efficient; we are seeing more compact housing and apartments to make the best use of available space. This does mean that large houses come at a premium.

The green corridor network has also meant less cars in the city. A number of key routes in the centre and connecting suburbs have become safe green lanes for walking and cycling only. This has encouraged more cycling and walking. The constraints on cars have also boosted public transport use and use of cargo bikes.

Overall, we are living much more with nature, wildlife populations have massively increased in the city, and the city is cleaner and more resilient to flooding and heat waves.

Table S1: Possible actions in future visions

		Mentioned by Living …			
**PHYSICAL ACTIONS**	**Description**	**In**	**From**	**With**	**As**
1. Community orchards	Communities are supported to plant and manage fruit and nut trees in publicly accessible greenspaces.				
2. Small community woodlands	Communities are supported to plant and manage woodlands focused on recreation and wellbeing in publicly accessible greenspaces.				
3. Promoting trees in private gardens	Households are supported to plant trees in their private gardens, helped by local government, social enterprises, and charities.				
4. Green roofs and rooftop gardens	Green roofs and rooftop gardens including appropriately sized trees are introduced on new developments by default, and retrofitted where possible on existing buildings.				
5. Tree-lining streets prioritised over parking and utilities	Trees are prioritised in the layout of streets, pavements and under-street utilities like broadband cables. This makes more space for trees but can reduce parking and mean moving utilities from under the pavement to under the road, which can mean more disruption to transport due to maintenance.				
6. Change minor streets to pedestrian and cycle zones, making more space for trees	More space for trees is created by increasing the number of minor and residential roads that prioritise walking, cycling and recreation.				
7. Large national woodland reserves outside city	Existing woodlands are expanded and connected, and new woodlands are planted, with a focus on creating large, interconnected reserves for nature conservation.				
8. Agroforestry on farms around city	Agroforestry is the integration of trees into farming systems while maintaining agricultural production. Farms are encouraged to plant lines of trees on fields, so that they have trees alongside livestock or crops, including fruit and nut trees.				
9. Tree planting for flood prevention	Tree planting is targeted towards encouraging rainwater to soak into the soil, for example through reducing paving around trees, and choosing species that best slow down the flow of water.				
10. Green corridors / arteries	Tree planting is targeted to connect existing and new green spaces, for example through 'pocket parks' and hedgerows, providing green space for people and allowing wildlife to move through the city better.				

		Mentioned by Living …			
**SOCIAL AND LEGAL ACTIONS**	**Description**	**In**	**From**	**With**	**As**
1. Right to access to greenspace within 200m of every house	The local authority has an obligation and plan to ensure access to half a football pitch (0.5ha) of greenspace (including trees) within a five-minute walk (200m) of every home.				
2. Increased green prescriptions and green spaces at medical and care facilities	NHS trusts/health boards enact policies that encourage and support GPs to prescribe participation in outside activities for preventative and restorative mental and physical health. NHS and care sector develop policies to ensure accessible green spaces at care facilities wherever possible.				
3. Increased ecotherapy and shift of health/wellbeing activities from indoor to outdoor	Local charities (e.g. environment, health, aging, disability etc.) expand their range of, and shift their focus to outdoor therapeutically oriented activities.				
4. Tree for every child	Local councils work with environmental charities to make a tree available for planting in a garden or public place, for every child born.				
5. Policy for child engagement with trees and outdoor education in every school	Connection with nature becomes a central element of all child-related policies and becomes a central element of the curriculum, such as through forest schools in every primary school and bushcraft in every secondary school.				
6. Trees have legal guardians	Trees receive a legal right to be represented in decision making and court, supported by a register of volunteer citizen guardians.				
7. Green culture and business development plan to take more advantage of green spaces	Local government, businesses, and arts and culture organisations work together on a development plan to better harness and develop greenspaces and trees for economic opportunities (e.g. tourism, hospitality, media, outdoor performance)				
8. Very strong protection for veteran and heritage trees	Veteran (very old) and culturally significant (e.g. memorial) trees are protected from development.				
9. New developments have biodiversity as a hard constraint	Developments are not allowed to have a significant negative impact on local biodiversity (the diversity of habitats and species present) or rare or threatened wildlife. This means developments generally only take place on brownfield or low-biodiversity agricultural land.				
10. Biodiversity loss by developments can be compensated elsewhere	Developments are allowed to have a significant negative impact on local biodiversity (the diversity of habitats and species present), but the impacts must be compensated by creating extra nature elsewhere (for example, on lower cost land further away from the city).				

		Mentioned by Living …			
**DECISION MAKING AND MANAGEMENT RESPONSIBILITY**	**Description**	**In**	**From**	**With**	**As**
1. **Community groups** have key influence on design, species selection, and management decisions	Local communities play a central role in making decisions. There is extensive public participation in designing the future of the treescape and extensive volunteering in the upkeep of trees and green spaces.				
2. **Professional tree experts** have key influence on design, species selection, and management decisions	Professional tree experts play a central role in decisions, with more limited participation for local communities.				
3. **Markets and companies** have key influence on design, species selection, and management decisions	Companies and investors play a central role in deciding what the treescape is for. Expansion and maintenance of the treescape is oriented towards business, whether through schemes like carbon markets and biodiversity banking, or increasing commercial benefits such as increasing visitor numbers by planting trees along high streets.				

Text S2: York Vision of a future treescape

Text is drawn from synthesis of the four Tree Value Visions according to priority outcomes and actions set by the York Citizen Panel, with added verbatim quotes from citizens' stories of the future (marked as underlined).

Local residents are meeting to celebrate 25 years since they planted a community orchard together. A child plays with his friends among the blossoms. The orchard, which the child's mother remembers planting when she was a teenager as part of an educational program, now produces fruit that is shared as part of the celebrations.

In York, 50 years ago, citizens came together to develop a common vision of future treescapes, much of which has now been realised. Citizens' top priorities included improving health and wellbeing, equity in access, biodiversity, environmental education, local food production, and regulating ecosystem services (for example, the ways that treescapes help to slow stormwater to prevent floods, protect us from extreme heat and regulate our local and global climate). Increasing the number of small green spaces in the city was core to their vision.

Improving our health and wellbeing involved everyone, from businesses, to the health service and community members. We focused on creating small green spaces for everyone to recreate and meet up with each other, and escape the stresses of life and the heat waves that we get more often now due to climate change. Many people find peace and belonging here and this benefits our mental and physical health.

Green prescriptions are strongly embedded in the health service and there are policies in place that ensure that all hospitals and care homes look out onto green space, speeding up recovery.

Due to new local government policy, everyone in our city now has access to a woodland or park within 300 metres of their home. The inequality that used to be there, where some people would struggle to find accessible green space, is gone.

The small green spaces that help achieve this take many forms: community orchards; a seating area on a street corner or event space where there used to be a derelict site, surrounded by trees; an area of trees in a school field; and many small mixed-age community woodlands dotted around the city that feel like a natural part of the community. Some of the community woodlands are memorial woodlands, where a tree is planted for everyone who dies in the city. The woodland becomes a special place for their family to visit and remember them. In the centre of York, the trees are full of long tailed tits and wag tails, the canopy is full of life with butterflies, bees and other insects buzzing and adding to the music in the marketplace.

Many of the smaller green spaces are managed decentrally through neighbourhood groups, supported by the council's tree officers. The tree species planted are prioritised by local people, and many communities choose trees that provide colours in the autumn and blossom in the spring. Climate resilience has also been important and we selected species for drought and flood resilience in collaboration between experts and the community.

Nature bridges connect green spaces across main roads, street corners and derelict spaces have been revived into pocket parks or tiny forests, with information boards on the history of the treescape and biodiversity that the spaces provide. Small green spaces are linked up where possible, including through pocket parks, tree lined avenues, and safe green lanes for walking and cycling only, encouraging active travel. Rooftops have also become homes for trees and the city issued a planning requirement that all new developments and existing streets must be treelined unless there are strong overriding impediments.

This way, the city has become a living landscape that has reduced flood risk, added shade and colour, urban cooling and cleaner air and brought an abundance of birds, insects and other wildlife. Tree planting maximises connectivity and biodiversity, with a recognition that trees and the animals and plants that depend on them deserve to be protected for their own sake. Planning in the connected, living landscape has biodiversity as a hard constraint: developments cannot go ahead if they have a significant negative effect on biodiversity or rare species. This has meant that we have had to be very selective in terms of where we've been able to expand housing and other

development, restricting it to brown field sites and low-grade agricultural land. Thus large houses come at a premium and there are more flats.

Perhaps most important of all, we developed a policy to maximise educational engagement throughout people's lives. School children engage with trees through planting and pruning trees for tree climbing, den making, foraging and other sensory activities. Forest schools are embedded in every primary school and bushcraft in every secondary school curriculum, supporting parents, and ensuring inclusivity for those with additional learning needs. Many employers in the city now run programmes to support employee wellbeing by providing opportunities to learn the skills to care for the trees as well as providing green views to reduce stress and increase productivity. Agroforestry farms on the edge of the city have provided a new set of career opportunities which local colleges provide training in.

Overall, this led to quite an organic way in which we met government targets for tree planting. What once were derelict areas are now places full of new life. Not only is nature thriving, but people are also given new spaces to socialise and enjoy nature in their own way, with more opportunities for those who had been socially excluded. Our relationships changed – with the trees, with nature, with each other and with ourselves – and we became healthier physically and mentally through nature connection and being outside, more community focused, more sociable, more creative, and with much happier kids.
